# Flexible nailing: Pushing the indications for diametaphyseal lower-extremity fractures

**DOI:** 10.1097/MD.0000000000037417

**Published:** 2024-03-15

**Authors:** Gregory Benes, Jessica Schmerler, Andrew B. Harris, Adam Margalit, Rushyuan Jay Lee

**Affiliations:** a Johns Hopkins University Department of Orthopaedic Surgery, Baltimore, MD

**Keywords:** diametaphyseal, femur, fixation, fracture, tibia

## Abstract

Elastic stable intramedullary nailing (ESIN) has been shown to be an effective form of surgical management for lower-extremity diametaphyseal fractures in pediatric patients, but studies are limited because ESIN treatment for these fractures is relatively uncommon. We sought to determine whether ESIN can be used effectively in the most distal or proximal short-segment forms of these fractures. We queried the electronic medical record system at Johns Hopkins Hospital using Current Procedural Terminology codes for femur and tibia fractures treated with ESIN in patients under 18 years old between January 2015 and October 2022. Preoperative and postoperative radiographs were subsequently reviewed to identify patients with a proximal or distal third femoral or tibial shaft fracture treated with ESIN and to define criteria for short-segment diametaphyseal fractures. We used Beaty radiological criteria to evaluate radiographic outcomes and Flynn titanium elastic nails (TENs) outcome scale to assess clinical recovery after radiographic evidence of union. There were 43 children who met the inclusion criteria. Among them, 10 patients had short-segment diametaphyseal fractures. There were 22 (51.2%) who sustained femur fractures and 21 (48.8%) who sustained tibia fractures. Using Beaty radiologic criteria, ESIN was associated with more satisfactory outcomes in patients with distal or proximal third shaft fractures (32/33) than in patients with short-segment diametaphyseal fractures (7/10) (*P* = .03). Using the TENs outcome scale, 21 (63.4%) patients with distal or proximal third shaft fractures had excellent results, 11 (33.3%) had satisfactory results, and 1 (3%) had a poor result. Among patients with short-segment diametaphyseal fractures, 4 (40%) had excellent results, 5 (50%) had satisfactory results, and 1 (10%) had a poor result. There were no differences in TENs outcomes between the groups (*P* = .24). Patients with short-segment lower-extremity diametaphyseal fractures treated with ESIN had worse radiographic outcomes but did no worse clinically than patients with distal or proximal third shaft fractures. Consequently, ESIN should be considered a safe and effective surgical management option for pediatric patients with even the most distal or proximal forms of these fractures.

## 1. Introduction

Lower-extremity diametaphyseal fractures are relatively uncommon in pediatric patients, with subtrochanteric femur fractures accounting for 4%–10% of pediatric femoral fractures and distal tibial metaphyseal fractures accounting for only 0.35%–0.45% of all pediatric fractures.^[1,2]^ Subtrochanteric femur fractures have a tendency to be displaced and difficult to reduce because of deforming forces from the surrounding muscles.^[3,4]^ Displaced distal tibial metaphyseal fractures often present with valgus recurvatum or varus procurvatum, necessitating careful individualization of treatment and fixation.^[5]^ Furthermore, displaced fractures at these sites can be challenging to treat, particularly in the subtrochanteric region of the femur, which has low remodeling capability.^[3,6]^ Consequently, although the majority of pediatric lower-extremity fractures are treated conservatively with immobilization, surgery including intramedullary nailing and submuscular plating is typically indicated in the case of displaced fractures.^[2,3]^ However, there are several notable shortcomings to these nonoperative and operative treatment modalities. For instance, prolonged cast immobilization can be complicated by loss of reduction as well as muscle atrophy, pressure sores, and thermal injury upon removal of cast.^[7,8]^ Additionally, in submuscular plating, hardware removal may be necessary due to prominence or irritation, which may have an increased refracture risk following hardware removal.^[9]^

Elastic stable intramedullary nailing (ESIN) is considered the gold standard for surgical management of femoral and tibial shaft fractures.^[10–12]^ The minimally invasive surgical technique, first described by Métaizeau in 1982,^[13]^ involves advancing C-shaped elastic nails through the fracture site for osteosynthesis, which yields shorter hospital stays and a reduced chance of bacterial contamination.^[14]^ In recent years, several studies have examined the use of ESIN in distal diametaphyseal fractures in the lower extremities.^[2,3,6,12,15–18]^ Although they have had small sample sizes, the studies have demonstrated that ESIN is a safe and effective surgical management option for these fractures.^[2,3,6,12,15–18]^ However, they have primarily included patients with fractures in the more proximal portions of the diametaphyseal region, and controversy remains over whether ESIN can be used for fixation of the most distal and proximal diametaphyseal fractures.

To our knowledge, no studies have evaluated the use of ESIN in the most distal or proximal short-segment lower-extremity diametaphyseal fractures. This study aimed to fill this gap in the literature by examining radiographic and clinical outcomes in pediatric patients with short-segment subtrochanteric femur or distal tibial diametaphyseal fractures surgically managed with ESIN and comparing them with outcomes in patients with proximal or distal third shaft fractures treated with ESIN.

## 2. Methods

### 2.1. Patient population

After obtaining approval from the Johns Hopkins Medicine Institutional Review Board, we queried the institution’s electronic medical record system for femur and tibia fractures treated with intramedullary nailing based on Current Procedural Terminology codes 27506 and 27759, respectively, between January 2015 and October 2022. The initial query retrieved 532 patients younger than 18 years old who sustained a femur or tibia fracture and were treated with intramedullary nail fixation. Preoperative and postoperative radiographs of the 532 patients were subsequently reviewed to identify those whose fractures were in the proximal or distal third femoral or tibial shaft and were managed with ESIN. All of the surgical procedures were performed by 6 pediatric orthopedic surgeons. Closed physes did not preclude treatment with ESIN.

Sex, age at time of injury, mechanism of accident, laterality, initial displacement, presence or absence of associated neurovascular injury, nail size, whether the fracture was open or closed, length of surgery, and postoperative complications were collected from the charts. Anteroposterior (AP) and lateral radiographs were reviewed to classify fractures as oblique or transverse.

### 2.2. Short-segment fractures

**Figure 1.**
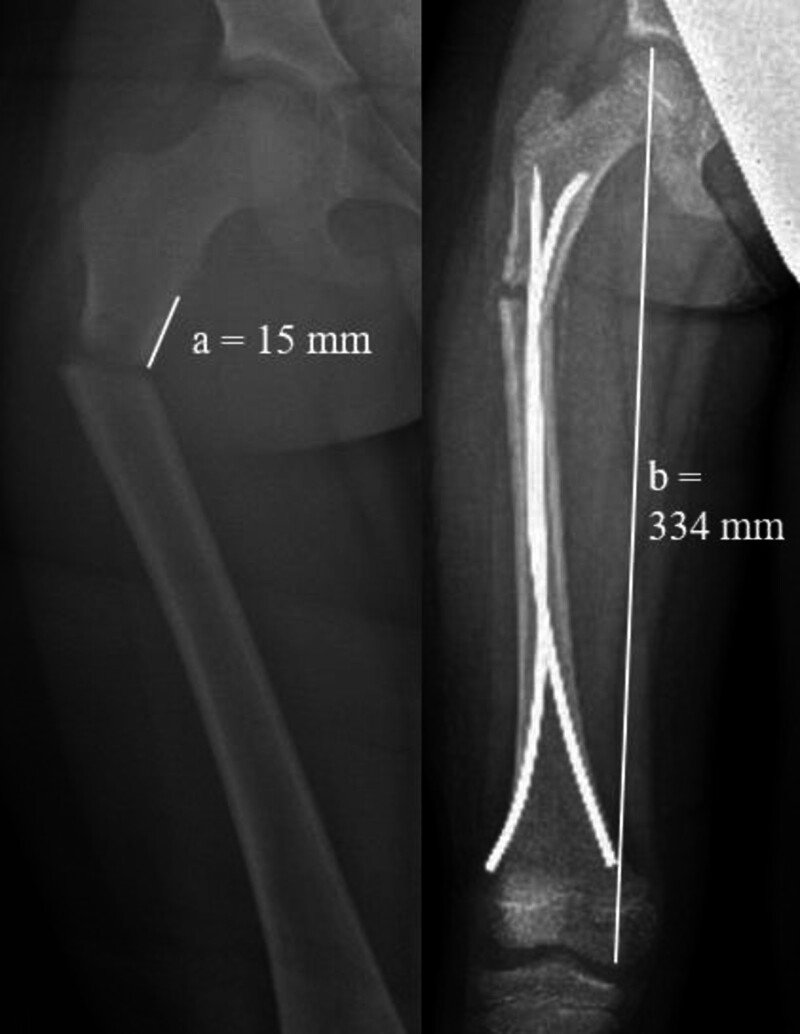
Radiographs demonstrating the method used to define short-segment subtrochanteric femur fracture, with (A) representing the distance between the inferior aspect of the less trochanter and the fracture site and (B) representing the total femur length, defined as the distance between the top of the femoral head and the medial femoral condyle. If (a/b) × 100 = <5%, the fracture was classified as short-segment. In this case, a = 15 mm, and b = 334 mm; 15/334 × 100 = 4%.

**Figure 2.**
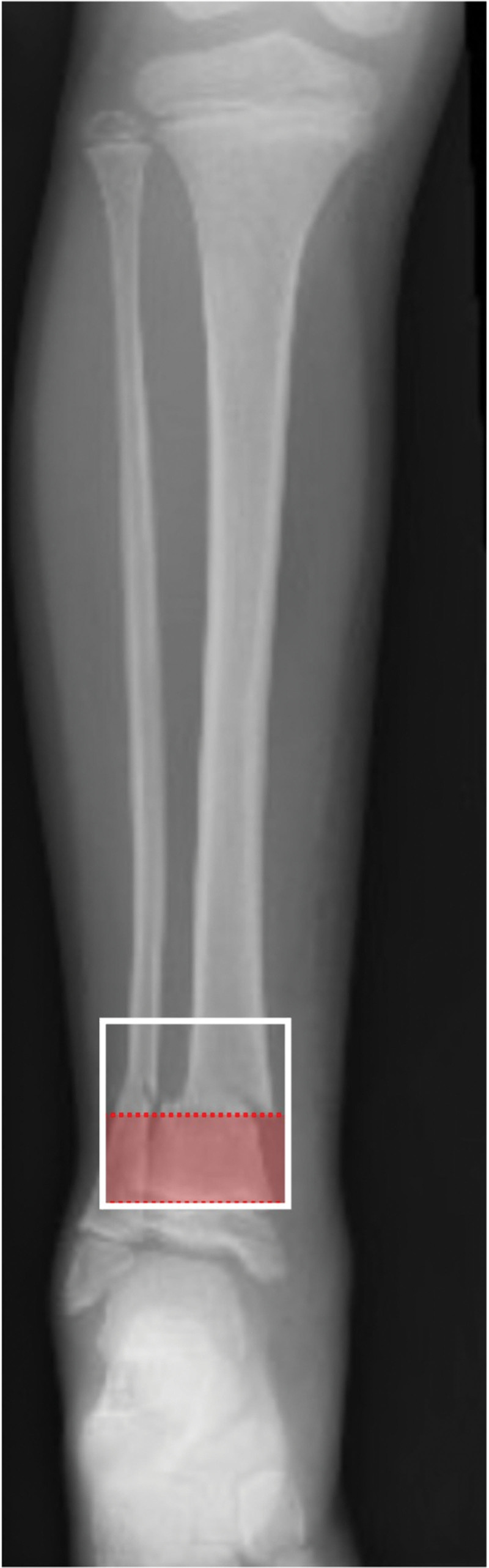
Radiograph showing fracture location area for short-segment diametaphyseal fractures. The white box is the epiphysis. The red shaded box, which is 50% of the width of epiphysis, is the defined location for short-segment diametaphyseal fractures.

Previous literature has defined a subtrochanteric fracture as a fracture occurring within 10% of the total femur length below the lesser trochanter^[12,19]^; therefore, we defined short-segment subtrochanteric femur fracture as a fracture located within 5% of the total femur length below the lesser trochanter. The first available postoperative full-length AP femur radiograph was used to determine the total length of the femur, which was defined as the distance between the top of the femoral head and the medial femoral condyle.^[12]^ The distance between the inferior aspect of the lesser trochanter and the fracture site was measured. If this distance was <5% of the total length of the femur, the fracture was classified as short-segment subtrochanteric (Fig. 1). Additionally, previous studies have defined distal tibia diametaphyseal fractures as those that occur in the square surface of bone of height equal to the widest portion of the epiphysis, the distal end being the physeal plate.^[2,5,20]^ Our aim was to look at shorter-segment fractures, which we defined as fractures occurring within the square surface of bone height equal to 50% of the widest portion of the epiphysis (Fig. 2).

**Figure 3.**
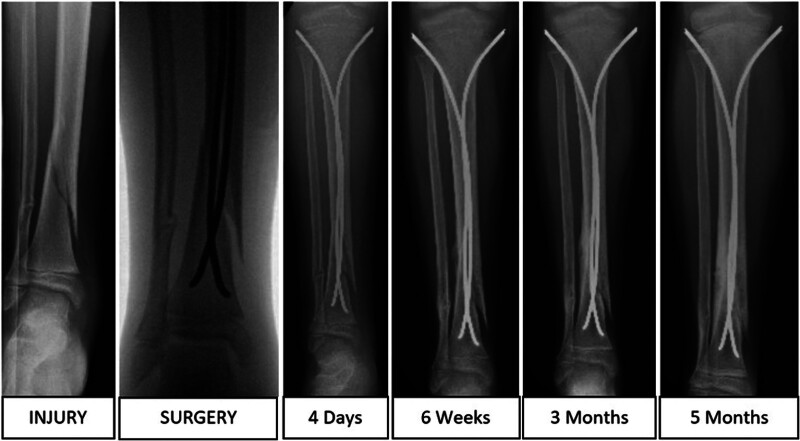
Anteroposterior radiographs of 6-year-old female patients with diametaphyseal fracture of right distal tibia with 6° of valgus on final radiograph prior to implant removal.

Patients were included if they had fractures that met these criteria, underwent definitive surgery with ESIN, and had complete clinical and radiographic follow-up until fracture union, defined as radiographic bridging callus across at least 3 of the 4 cortices (Fig. 3).^[21]^

### 2.3. Outcomes

Initial varus or valgus displacement and amount of recurvatum or procurvatum were assessed on AP and lateral radiographs, respectively. Fracture malalignment was determined based on review of radiograph at latest follow-up, and the greatest malalignment was recorded. Malunion was defined as varus or valgus >10° or anterior bowing >15°.^[22]^ Leg length discrepancy (LLD) was determined based on clinical exam or full-length bilateral lower-extremity radiographs, if available. Presence of pain was defined as consistent complaints of pain, whereas absence of pain was defined as absence of reports of pain or reports of intermittent pain.^[12]^ A major complication was defined as any complication that led to unplanned surgery. Minor complications, such as fracture malalignment or LLD, were defined as complications that resolved with nonoperative treatment or did not require any treatment. Beaty radiological criteria were used to evaluate the radiographic outcome.^[23]^ Flynn titanium elastic nails (TENs) outcome scale was used to assess clinical recovery.^[24]^ Fracture malalignment ≥6° and leg-length inequality ≥1 cm were considered minor complications if they did not lead to unplanned surgery, as these criteria denote a “satisfactory” result rather than an “excellent” result in the TENs outcome scoring system.^[12,24]^

### 2.4. Statistical analysis

Student *t* tests were utilized to compare outcomes measures between the short-segment and distal third fracture groups. A value <0.05 was considered significant.

## 3. Results

**Figure 4.**
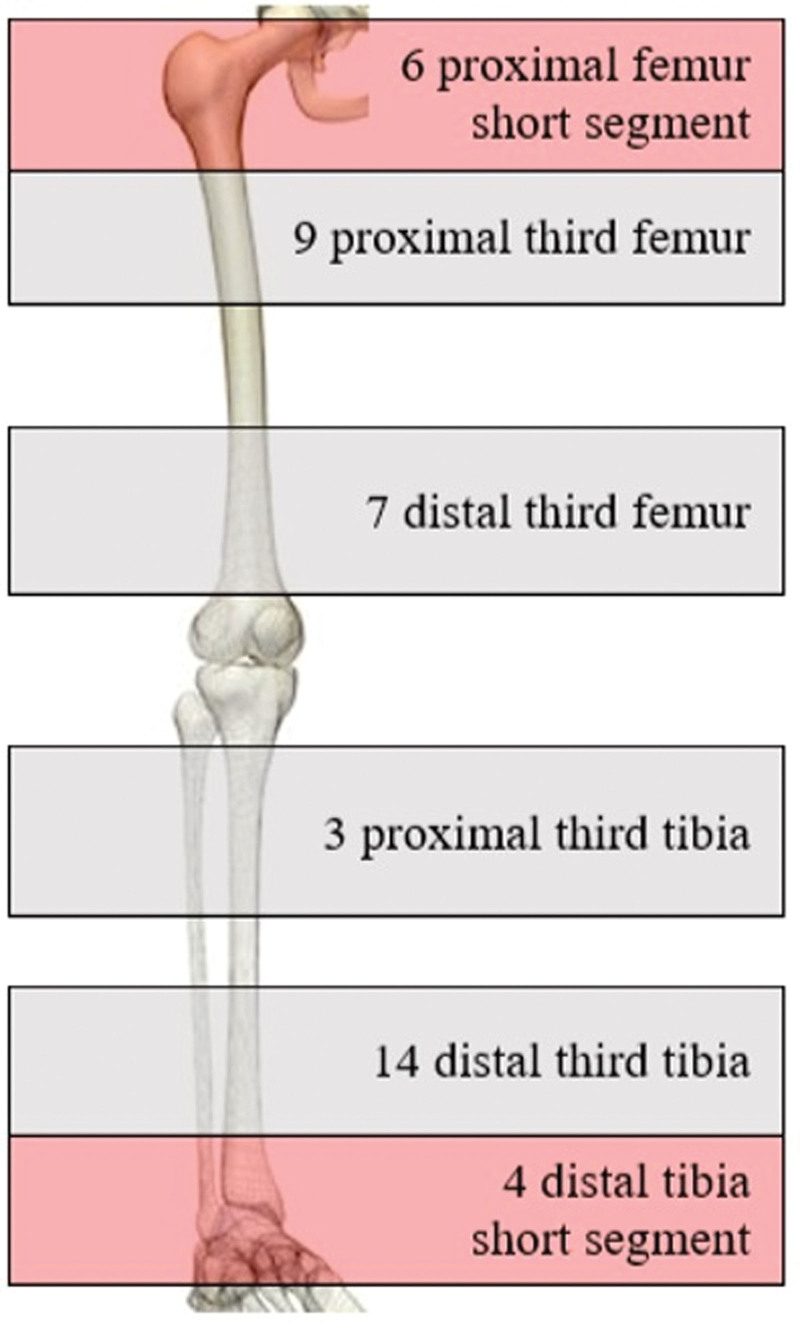
Anatomic distribution of fracture location among cohort of 43 pediatric patients.

A total of 43 children with a displaced proximal or distal third shaft fracture of the femur or tibia without neurovascular compromise met the inclusion criteria and were treated by ESIN (Fig. 4). Ten of the patients were classified as having short-segment diametaphyseal fractures.

Among the 43 patients, there were 28 boys (65.1%) and 15 girls (34.9%), with a mean age at fracture of 9.8 ± 3.5 years (range 5–15 years) (Table 1). The mechanisms of injury involved direct trauma, including car accidents (*n* = 18, 41.9%), being struck by a vehicle as a pedestrian (*n* = 6, 14%), and ballistic incidents (*n* = 1, 2.3%), and indirect trauma, including falls (*n* = 12, 27.9%) and sports-related injuries (*n* = 6, 14%). No pathologic fractures occurred. Twenty-two patients (51.2%) sustained femur fractures and 21 patients (48.8%) sustained tibia fractures. The affected side was the right extremity in 29 cases (67%) and the left extremity in 14 cases (33%).

**Table 1:** Demographic and fracture data for 43 patients with short-segment diametaphyseal and third shaft fractures.

	Short-segment diametaphyseal fracture group (*n* = 10)	Third shaft fracture group (*n* = 33)	Total (*n* = 43)	*P*
Age	7.8 ± 2.8	10.4 ± 3.4	9.8 ± 3.5	.02*
Sex				
Female	4	11	15	.49
Male	5	22	28	
Weight (kg)	35.7 ± 16.6	41.9 ± 17.7	41.1 ± 17.4	.07
BMI (kg/m^2^)	21.6 ± 2.8	18.4 ± 2.8	18.6	.07
Fracture location				
Femur	6	16	22	.39
Tibia	4	17	21	
Fracture pattern				
Oblique	3	17	20	.20
Transverse	7	16	23	
Fracture type				
Closed	9	28	37	.57
Open	1	5	6	
Laterality				
Left	2	12	14	.29
Right	8	21	29	

* *P* < .05.

There were 23 transverse-type fractures (53.5%) and 20 oblique-type fractures (46.5%). Among the 43 fractures, 9 had varus-procurvatum deformity (20.9%), 5 had valgus-procurvatum deformity (11.6%), 5 had varus-recurvatum deformity (11.6%), 5 had valgus-recurvatum deformity (11.6%), 5 had procurvatum-only deformity (11.6%), 6 had recurvatum-only deformity (14%), 6 had valgus-only deformity (14%), and 2 had varus-only deformity (4.7%).

Of the 10 patients with short-segment fractures, 3 had oblique and 7 had transverse fracture patterns. Within this group, there was 1 patient (10%) with an open fracture.

Among the 43 fractures, 21 were treated with 4 mm nails, 6 were treated with 4.5 mm nails, 7 were treated with 3.5 mm nails, 7 were treated with 3 mm nails, 1 was treated with a combination of 3 and 3.5 mm nails, and 1 was treated with 2.5 mm nails. Mean surgical time was 73 minutes (range 35–127 minutes). The average time to implant removal was 17 months (range 8–32 months). All patients returned to their previous daily activities, including sports, without delay, discomfort, or difficulty. At latest follow-up, 1 patient had a 1-cm LLD (injured extremity longer) noted on clinical exam after proximal femur diametaphyseal fracture, and 2 patients had discrepancies of 1.3 and 1.7 cm (injured extremities longer), respectively, measured on leg length radiographs after distal tibial diametaphyseal fracture and proximal third femur fracture. No surgical interventions were required for treatment of LLD.

Complications and radiographic outcomes for the short-segment diametaphyseal fractures are shown in Table 2. According to Beaty radiologic criteria, distal or proximal third shaft fractures were more often associated with satisfactory outcomes (32/33) than were short-segment diametaphyseal fractures (7/10) (*P* = .03). Using the TENs outcome measure scale, 21 (63.4%) patients with distal or proximal third shaft fractures had excellent results, 11 (33.3%) patients had satisfactory results, and 1 (3%) patient had a poor result. By comparison, 4 (40%) patients with short-segment diametaphyseal fractures had excellent results, 5 (50%) patients had satisfactory results, and 1 (10%) patient had a poor result. There were no differences in ESIN outcomes between the groups (*P* = .24).

**Table 2:** Complications and radiographic outcomes among 10 patients with short-segment diametaphyseal fractures.

Patient	Fracture location	Malalignment	Degree (°)	Leg length discrepancy (cm)	Pain	Complication	Beaty criteria	TENs outcome score
1	Femur	Valgus	7	No	No	Minor and resolved	Poor	Satisfactory
2	Tibia	Valgus	6	No	No	Minor and resolved	Poor	Satisfactory
3	Tibia	Valgus	4	No	No	No	Satisfactory	Excellent
4	Tibia	Recurvatum	4	No	No	No	Satisfactory	Excellent
5	Tibia	None	0	1.3	No	Minor and resolved	Satisfactory	Satisfactory
6	Femur	Recurvatum	15	No	No	Malunion	Poor	Poor
7	Femur	Procurvatum	9	No	No	Minor and resolved	Satisfactory	Satisfactory
8	Femur	Recurvatum	4	No	No	No	Satisfactory	Excellent
9	Femur	Valgus	4	No	No	No	Satisfactory	Excellent
10	Femur	Procurvatum	3	1.0	No	Minor and resolved	Satisfactory	Satisfactory

## 4. Discussion

This study sought to determine whether ESIN can be used effectively in the most distal or proximal short-segment forms of lower-extremity diametaphyseal fractures. ESIN has traditionally been the surgical management option of choice for femoral and tibial shaft fractures,^[10–12]^ but its use in distal or proximal diametaphyseal fractures has been controversial. These fractures are difficult to treat and they pose challenges to operative fixation, so they have historically been treated with cast immobilization or plating.^[10,17,25]^ However, as casting has been shown to be associated with poor radiographic outcomes, redisplacement in cast, and delayed return to activities,^[12,26]^ ESIN and other options for surgical management, such as compression plating, have been proposed. Recent literature has provided evidence that ESIN can be used safely and effectively in distal and proximal diametaphyseal fractures of the tibia and femur,^[2,3,6,12,15–18]^ but these studies have been limited by small sample sizes and have left unanswered the question of whether this technique can be used in the most distal and proximal fractures. The results of our study demonstrate that although radiographic outcomes after ESIN in short-segment fractures were inferior to those in distal or proximal third shaft fractures, clinical outcomes were no different, suggesting that ESIN can be considered as a management option in these difficult-to-treat fractures.

The finding that ESIN is safe and effective in short-segment fractures represents an addition to the literature showing promising results in the use of ESIN for subtrochanteric femur fractures. Although numerous studies have shown that ESIN can be used safely in pediatric patients with subtrochanteric femur fractures,^[3,12,17,18,27]^ providing support for this minimally invasive technique relative to traditional plate fixation, some studies have reported that ESIN is associated with a higher complication rate than plate fixation in these fractures.^[12,17]^ Xu et al^[17]^ suggested that ESIN may be more suitable in younger, lighter-weight children. In our study, all but 1 patient undergoing ESIN for short-segment subtrochanteric femur fracture had a satisfactory or better TENs outcome score. Notably, the patient with the poor outcome in this group had the greatest degree of fracture malalignment at baseline. Additionally, risk of growth disturbance is an important consideration. There was 1 patient, who sustained a short-segment proximal femur fracture, who was clinically noted to have an LLD of 1 cm overgrowth lengthening of the fractured limb. No further surgical interventions were required. Consequently, ESIN may be considered a safe and effective surgical management option for pediatric patients with short-segment subtrochanteric femur fractures within a reasonable degree of baseline fracture malalignment. Future work should investigate whether there are thresholds of fracture malalignment beyond which ESIN is no longer effective. Furthermore, as our study primarily assessed clinical and radiographic outcomes, future studies should evaluate factors associated with complications in order to more optimally anticipate which patients will benefit most from ESIN rather than plate fixation.

The literature evaluating ESIN in distal tibial metaphyseal fractures is even more limited, and the use of ESIN in these cases has been controversial because the proximity of the fractures to the ankle complicates the surgical technique and makes optimal stability of the nails difficult to achieve.^[15]^ Cravino et al^[2]^ examined 18 pediatric patients who underwent ESIN for distal tibial metaphyseal fractures, and all patients had positive radiographic outcomes, were pain-free at last follow-up, and regained normal activity levels. Pogorelić et al^[16]^ found that among 21 patients with distal tibial diametaphyseal fractures and 111 with tibial shaft fractures, those with distal fractures had a slightly higher rate of complications (14.3% vs 10.8%). Finally, Shen et al^[15]^ reported that among 21 patients with distal tibial diametaphyseal fractures, 19 (90%) showed radiographic and clinical healing at 5 months and all had full range of motion at the knee and ankle joints at final follow-up. However, all patients in the study had fractures at the proximal component of the distal tibial diametaphyseal junction, and the authors assert that ESIN at the distal component of the junction (akin to the short-segment fractures assessed in our study) would be too difficult and too unstable without anchoring the nails in the epiphysis, thereby disturbing the growth plate. The results of our study provide evidence to the contrary, demonstrating not only that ESIN in the distal short-segment of distal tibial diametaphyseal fractures leads to similar clinical improvement relative to distal third tibial shaft fractures managed with ESIN, but also that growth plate disturbance is a minimal risk with only 1 patient among the distal tibial diametaphyseal group experiencing a 1.3 cm LLD with overgrowth of the involved extremity. This LLD was likely due to overgrowth healing in pediatric fractures and not secondary to growth disturbance. No interventions were required for management of LLD.

Although the results of this study represent a valuable addition to the literature on ESIN management of distal lower-extremity diametaphyseal fractures, several limitations should be considered. First, as this was a retrospective study, the outcomes could have been influenced by factors not controlled for in this analysis as well as selection bias for treatment decision. Second, although the study controlled for certain demographic and fracture characteristics, patients in the 2 groups were not matched on these characteristics, which may have impacted the results. Third, the analysis should be interpreted in light of a small sample size of only 10 short-segment fractures. Future work should endeavor to prospectively follow a larger sample of patients with short-segment fractures and a cohort of matched controls. Finally, although the follow-up period in this study extended through complete radiographic union, it was not long enough to state with certainty that growth plate disturbance did not occur in patients.

## 5. Conclusions

In summary, the most distal and proximal short-segment lower-extremity diametaphyseal fractures can be extremely challenging to treat, and it has been debated whether ESIN is an acceptable form of surgical management for patients with these fractures. In this study, patients treated with ESIN after sustaining short-segment fractures had worse radiographic outcomes, predominantly because of malalignment, but no difference in clinical outcomes when compared with patients who had ESIN for proximal or distal third shaft fractures. Additionally, no patient in the short-segment fracture cohort experienced shortening of the fractured limb due to a growth disturbance. All observed LLDs occurred because of fracture overgrowth. Future research should examine long-term complications such as growth plate disruption in a larger patient sample size, to establish more concretely the safety and efficacy of ESIN in short-segment fractures.

## Acknowledgments

For editorial assistance, we thank Sandra Crump, MPH, in the Editorial Services group of The Johns Hopkins Department of Orthopaedic Surgery.

## Author contributions

**Conceptualization:** Gregory Benes, Jessica Schmerler, Andrew B. Harris, Adam Margalit, Rushyuan Jay Lee.

**Data curation:** Gregory Benes, Rushyuan Jay Lee.

**Formal analysis:** Gregory Benes, Jessica Schmerler, Rushyuan Jay Lee.

**Investigation:** Gregory Benes, Jessica Schmerler, Andrew B. Harris, Adam Margalit, Rushyuan Jay Lee.

**Methodology:** Gregory Benes, Jessica Schmerler, Andrew B. Harris, Adam Margalit, Rushyuan Jay Lee.

**Project administration:** Gregory Benes, Andrew B. Harris, Adam Margalit, Rushyuan Jay Lee.

**Validation:** Gregory Benes, Jessica Schmerler, Andrew B. Harris, Adam Margalit, Rushyuan Jay Lee.

**Visualization:** Gregory Benes, Jessica Schmerler, Andrew B. Harris, Adam Margalit, Rushyuan Jay Lee.

**Writing—original draft:** Gregory Benes, Jessica Schmerler, Andrew B. Harris, Adam Margalit, Rushyuan Jay Lee.

**Writing—review & editing:** Gregory Benes, Jessica Schmerler, Andrew B. Harris, Adam Margalit, Rushyuan Jay Lee.

**Resources:** Andrew B. Harris, Adam Margalit, Rushyuan Jay Lee.

**Supervision:** Andrew B. Harris, Adam Margalit, Rushyuan Jay Lee.

**Funding acquisition:** Rushyuan Jay Lee.
